# Treatment of harmful gambling: a scoping review of United Kingdom-based intervention research

**DOI:** 10.1186/s12888-024-05843-8

**Published:** 2024-05-23

**Authors:** Christopher J. Seel, Matthew Jones, Darren R. Christensen, Richard May, Alice E. Hoon, Simon Dymond

**Affiliations:** 1 School of Psychology, Swansea University, Singleton Campus, Swansea, SA2 8PP UK; 2 Faculty of Health Sciences, University of Lethbridge, Lethbridge, AB Canada; 3 School of Psychology and Therapeutic Studies, University of South Wales, Pontypridd, CF371DL UK; 4 Swansea University Medical School, Singleton Campus, Swansea, SA2 8PP UK; 5 Department of Psychology, Reykjavík University, Menntavegur 1, Nauthólsvík, 101 Reykjavík, Reykjavik, Iceland

**Keywords:** Gambling, Treatment, Scoping review, United Kingdom

## Abstract

**Background:**

Understanding and treating the harm caused by gambling is a growing international psychiatric and public health challenge. Treatment of gambling harm may involve psychological and pharmacological intervention, in conjunction with peer support. This scoping review was conducted to identify, for the first time, the characteristics and extent of United Kingdom (UK) based gambling treatment research. We reviewed studies conducted among people seeking treatment for disordered or harmful gambling in the UK, the settings, research designs, and outcome measures used, and to identify any treatment research gaps.

**Methods:**

Systematic searches of PsycInfo, PsycArticles, Scopus, PubMed, and Web of Science databases were carried out for gambling treatment research or evaluation studies conducted in the UK. Studies were included if they evaluated the effectiveness of an intervention or treatment designed to improve symptoms of harmful or problematic gambling, reported outcomes of interventions on treatment adherence, gambling symptoms, or behaviours using standardised measures, were conducted in the UK, and were published since 2000.

**Results:**

Eight studies met the inclusion criteria. Four were retrospective chart reviews, two were single-participant case reports, one described a retrospective case series, and one employed a cross-sectional design. None used an experimental design.

**Conclusion:**

The limited number of studies included in this review highlights a relative paucity of gambling treatment research conducted in UK settings. Further work should seek to identify potential barriers and obstacles to conducting gambling treatment research in the UK.

**Supplementary Information:**

The online version contains supplementary material available at 10.1186/s12888-024-05843-8.

## Background

It is estimated that approximately half of the United Kingdom (UK) population has engaged in gambling in the past year [1]. The prevalence of those at risk of, or already engaged in, problem or harmful gambling may be between 0.4 and 2.8% of the UK population [2, 3]. Problematic patterns of gambling are characterised by a range of harms including financial difficulties, harms to health and well-being, relationship breakdown, and increased risk of suicidality [4, 5]. The increased availability of gambling is linked with higher rates of gambling problems [4, 6] and the harms caused by gambling are now considered to represent a global public health challenge [7–9].

Internationally, only one in five individuals with experience of problem or harmful gambling seek help [10]. In the UK, treatment for harmful gambling can involve a combination of professional- or self-referral to the National Gambling Support Network (NGSN) delivered by GambleAware, attending NHS gambling harms services (currently available in fifteen sites in England only), third sector treatment providers, and peer aid/mutual support groups. Following initial assessment and screening, clients may receive a Tier 2 referral which typically involve brief interventions (i.e., 1–3 sessions) including core principles of cognitive behavioural therapy (CBT) and motivational interviewing delivered by the NGSN or thirdsector providers. For instance, Addiction Recovery Agency (ARA) provides counselling and support services in Wales and the South West of England for people with drug and alcohol, mental health, and gambling related problems. Following a comprehensive assessment, clients may receive a Tier 3 referral to structured (i.e., 6–10 sessions) psychosocial interventions or to NHS-based service providers for those with more complex needs. For instance, the National Centre for Behavioural Addictions houses the National Problem Gambling Clinic in London, while the recently established NHS Northern Gambling Service and NHS Southern Gambling Service provide specialist treatment to people experiencing problems with gambling. Tier 4 referrals involve structured treatment delivered in residential care for those with evidence of severe harms from gambling. For instance, Gordon Moody and Adferiad organisations provide residential courses in England and Wales, respectively. Despite these examples, the gambling treatment options in the UK remain limited and has led to recent calls for greater investment in services [11, 12].

Evidence-based guidelines for the treatment of problem or harmful gambling within countries or jurisdictions, such as Australia, recommend individual or group CBT and motivational interviewing/motivational enhancement therapy to reduce gambling behaviour, severity, and psychological distress [13, 14]. In the UK, this would approximate a Tier 2 intervention, with extended duration structured treatment resembling a Tier 3 intervention. The guidelines recommend that practitioner-delivered psychological interventions be used rather than self-help strategies, antidepressant medication should not be prescribed in isolation, and that while pharmacological interventions may produce short-term changes in gambling symptom severity, further investigation is warranted before their efficacy is fully determined [15].

In England, the National Institute for Health and Care Excellence (NICE) is currently developing guidelines for the identification, assessment, and management of harmful gambling [NICE, 2022; 16]. The recommendations, which are due to be published in 2024, are intended to address “current gaps in care” such as “poor provision of treatments aimed at specific groups of people (for example, different age groups, different ethnic groups, and people with comorbidities) and a lack of follow-up and ongoing care.” (NICE, 2022, p.3). Moreover, “most treatments are offered on a short-term basis and relapse is common. There is also a lack of identification and support for other people affected by a person’s harmful gambling, such as family members, friends and others close to them.” (NICE, 2022, p.3). The settings covered by the proposed new guidelines will include “all settings where harmful gambling may be identified” and “settings where NHS-commissioned healthcare is provided for people who participate in harmful gambling.” (NICE, 2022, p.5). While the development and forthcoming publication of these treatment guidelines is welcome, it has been noted that there is a relative dearth of UK (i.e., England, Scotland, and Wales) based outcome research on gambling interventions [11]. Indeed, a systematic review of treatments for gambling published between 2005 and 2016 did not identify any UK-based studies [17], while a rapid evidence assessment report of treatment gaps for gambling noted that only 5% of identified studies were conducted in the UK [18]. To date, there has been no review of the gambling treatment literature specific to a UK-context.

The purpose of the current study was therefore to scope, for the first time, the characteristics and extent of UK-based gambling intervention research. Specifically, the current review sought to address the following research questions:

1. What gambling treatment evaluation studies have been conducted among people seeking treatment in the UK?
2. What are the settings, research designs, and outcome measures used to evaluate gambling treatments in the UK?
3. What are the gambling treatment research gaps?

## Methods

This scoping review was registered on the Open Science Framework (OSF; https://osf.io/bukhs) and conducted in accordance with both the PRISMA Extension for Scoping Reviews (PRISMA-ScR) [19] and the methodological framework outlined by Arksey and O’Malley [20]. We elected to conduct a scoping review rather than a systematic review as our goal was to identify the extant UK-based treatment evaluation research articles rather than make inferences concerning the feasibility or effectiveness of treatment approaches or practices [21].

### Data sources and search strategy

We conducted electronic literature searches of PsycInfo, PsycArticles, Scopus, PubMed, and Web of Science on January 13th 2023. The development of our search strategy was a collaborative effort and included consultation with an information specialist at Swansea University. The search strategy included the following terms: (gambl* OR betting OR wagering OR ludomania* OR ludopath*) AND (intervention* OR treatment* OR therap*) AND (UK OR “United Kingdom” OR “Great Britain” OR England OR Wales OR Scotland OR Ireland). Key words and Medical Subject (MeSH) headings were utilised where possible, except for terms related to location (i.e., “UK”) to maximise the likelihood of capturing UK-based studies. Corresponding authors of all studies included in our review as well as several UK-based gambling researchers were contacted by email to enquire if they were aware of any further studies that we may have omitted. No new articles were identified following this process.

### Eligibility criteria

Articles were eligible for inclusion if they (a) evaluated the effectiveness of a psychological, behavioural, or pharmacological intervention or treatment designed to improve symptoms of harmful gambling or evaluated moderators of treatment success, (b) reported outcomes of interventions on treatment adherence, harmful gambling symptoms, or behaviours using standardised measures, (c) were conducted in a UK-based setting or online with participants residing in the UK, (d) were published between January 1st 2000 and January 1st 2023, and (e) were peer-reviewed articles published in English. We excluded empirical articles that evaluated primary prevention (‘responsible’ or ‘safer gambling’) strategies [22], treatment evaluations where gambling behaviour or symptoms were not the primary outcome/dependent measure [23], literature or systematic reviews [17], and studies for which we were unable to obtain the full-text document.

Decisions regarding the location of the treatment study described in the article were determined via the following steps. First, the methodology was reviewed to identify whether the authors referred to either the study setting or the nationality of participants. If sufficient information was not provided to determine whether the study was conducted in the UK, we included studies if they either attained ethical approval from a UK-based institution or were written by an author or authors affiliated with a UK-based organization. In the event that a paper involved a collaboration between UK-based and international authors, we emailed the corresponding author for information regarding the study setting.

### Study selection

**Fig. 1:**
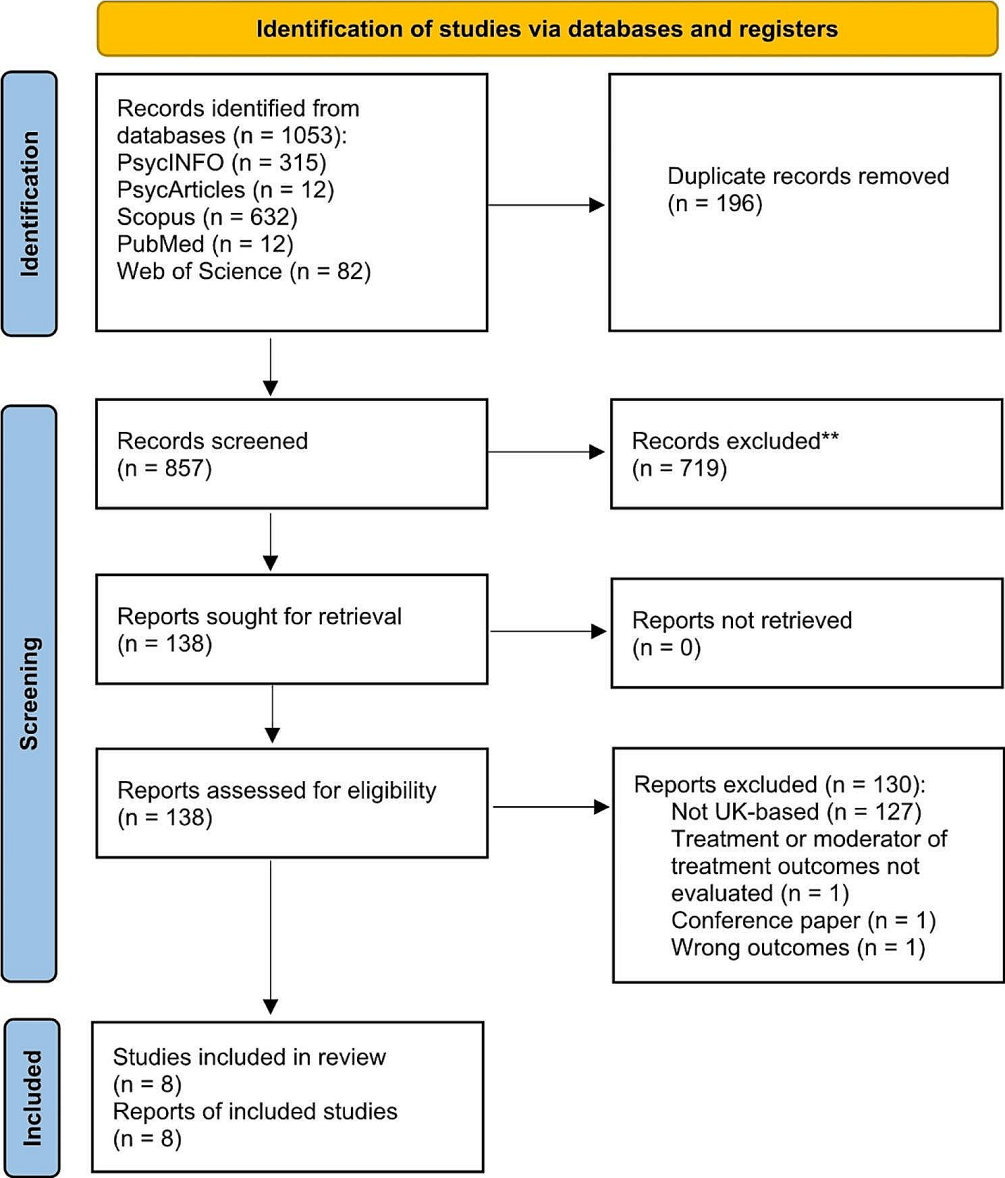
PRISMA diagram displaying results of search strategy

Search results were uploaded to Covidence, a web-based screening and data extraction software tool (https://www.covidence.org/), and duplicates removed (*n* = 196). Titles and abstracts were independently reviewed by CJS and MJ for inclusion. Inter-rater agreement was calculated by dividing the number of agreements by the number of agreements plus disagreements and multiplying by 100%. Agreement was 95.59% at this stage. The full texts of retained articles (*n* = 138) were uploaded to Covidence and were again independently reviewed by CJS and MJ. Inter-rater agreement at this stage was 99.3%. The one discrepancy between reviewers concerned whether a case report [24] should be included, given that authors only provided an informal comparison of pre-post gambling (i.e., did not systematically measure gambling symptoms or behaviour). After discussion, the study was included given that the study reported measures of real-world gambling (albeit informally). Our search strategy therefore resulted in 8 studies included in our review (see Fig. 1). Please see https://osf.io/6wbdm/ for the full list of excluded articles and reasons for exclusion.

### Data extraction and reporting

A data extraction form was created using Covidence. Two reviewers (CJS & MJ) independently piloted this form with two of the included studies to ensure it adequately addressed our research aims [25]. Following the initial data extraction, the reviewers resolved disagreements via discussion until consensus was reached. The research team then made some minor modifications to the data extraction form to increase the clarity of instructions. The two reviewers then extracted data from the remaining six included articles. We extracted: authors’ names, paper title, study funding information, number of participants, location and setting for study, intervention/treatment strategy, research design, comparator(s), recruitment strategy, duration of study, length of follow-up, outcomes measured, and study outcomes. Following extraction, data was collated, summarized, and reported using tables and narrative synthesis (see below). Risk of bias was not assessed given our aim was to provide an overview of the literature rather than formally evaluate the quality of the evidence-base [20, 25].

## Results

Basic characteristics (study designs, settings, participant information, etc.) of the eight included studies are presented in Table 1. The studies reported outcomes ranging from 18-months to three years except for Roberts et al. [26] which reported 16 years’ worth of clinical data. All but one of the studies [24] were published in the last decade, and none were published since the COVID-19 pandemic. Further characteristics of the included studies and main findings are summarised in Table 2.

**Table 1:** Characteristics of UK-based treatment outcome literature

Authors	Study design	Study setting	Sample size	Recruitment sources	Gender (M/F)	Age (M/SD, Range)
Bowden-Jones & George [24]	Case report	Gambling clinic (unspecified)	1	NS	1/0	62*
Hutchison et al., 30	Cross-sectional	Open GA mutual aid fellowship	44	Volunteers recruited on-site	41/3	49.72 (17.29)
Roberts et al., 26	Retrospective chart review	Both GMA sites	658	Clinic data	658/0	34.82 (9.98) 17–70
Ronzitti et al., (2015)	Retrospective chart review	NPGC	676	Clinic data	624/52	T cohort: 34.59 (10.03), NT cohort: 37.28 (11.59)
Ronzitti et al., (2018)	Retrospective chart review	NPGC	524	Clinic data	485/39	35.5 (10.7)
Ronzitti et al., (2017)	Retrospective chart review	NPGC	846	Clinic data	788/58	35 (10.37)
Shonin et al., (2014)	Case report	Not specified	1	NS	0/1	32*
Ward et al., (2018)	Retrospective case series	NPGC	10	Clinic data audit	8/2	44**

*Note* GA = Gamblers Anonymous, GMA = Gordon Moody Association, NPGC = National Problem Gambling Clinic, NS = not specified, NT cohort = No tobacco use, T cohort = tobacco use, NT cohort = no tobacco use. *n = 1. **Median reported

**Table 2:** Gambling-relevant study characteristics, including intervention type, and main findings of UK-based treatment outcome literature

Authors	Gambling status	Gambling-specific measures	Other measures	Intervention	Main findings
Bowden-Jones & George [24]	NS	Abstinence, cravings	None	CBT	Medication switch to rotigotine and 10 session CBT. Abstinence reported in 6 months post-treatment, but occasional cravings persisted.
Hutchison et al., [30]	NS	Abstinence self-efficacy, perceived risk	Recovery group identification, MSSS	GA – 12-step programme	Identification with recovery group predicted more perceived support, higher abstinence self-efficacy, and less perceived risk in gambling-related trigger situations. Relationship between recovery group identification and both abstinence self-efficacy and perceived risk mediated by the perceived provision of social support and not its receipt.
Roberts et al., [26]	PGSI; criteria NS	PGSI, gambling audit	Treatment completion, need audit, life audit	CBT	51.3% of sample dropped out. Age (over 26), higher education, higher debts, online gambling, gambling on poker, undertaking shorter treatment programme (3–6 months), depression, experience of previous treatment programmes and medication, and adverse childhood experiences predicted dropout. No difference between treatment completers and dropouts on PGSI scores. Enforced dropout predicted by gambling on sports events, smoking, and previous experience of homelessness or depression. Enforced dropout less likely for those in longer treatment programme (9 months) and those who had received any previous treatment.
Ronzitti et al., [31]	PGSI; score 3+	Gambling behaviour (interview), PGSI	PHQ-9, GAD-7, AUDIT-C, tobacco behaviour, drug use	CBT	46.4% of sample daily tobacco users. No significant difference in PGSI score or on clinical variables (GAD-7 and PHQ-9) between smokers and non-smokers, or on treatment completion and treatment outcome.
Ronzitti et al., [23]	PGSI; score 8+	PGSI, gambling behaviour (type, frequency, history, spendings, debts)	PHQ-9, GAD-7, AUDIT-C, tobacco & drug use	CBT	27.4% dropout pre-treatment and 17.4% of sample dropout during treatment. CBT format not related to dropout. Treatment dropouts more likely to be single, younger, unemployed, smoke, and score more highly on the PGSI, GAD-7, and PHQ-9 than completers. Pre-treatment dropouts linked to younger age and drug use; in-treatment dropouts linked to family history, lower PGSI score, and smoking.
Ronzitti et al., [32]	PGSI; score 8+	Gambling activities	Treatment completion	CBT	54.6% completed treatment, 20.8% dropout during, 24.6% dropout pre-treatment. Gambling on gaming machines predictor of pre-treatment dropout. Betting on sports events predicted treatment dropout.
Shonin et al., [27]	DSM-IV-TR	G-SAS, gambling frequency, duration, spend (daily diary)	BPRS, GAF, MAAS, GAS	2-phases: CBT then MAT	Moderate improvements during CBT treatment on BPRS and GAF but no difference on G-SAS or MAAS. Following MAT, significant improvement on all outcome variables. Abstinence achieved after MAT which maintained at 3-month follow-up.
Ward et al., [34]	PGSI; score 8+	GCS, gambling behaviour	Medication side effects	Naltrexone	6-week follow-up: All participants reported decreases in gambling cravings. 60% abstinent during treatment, and 20% reduced gambling to low levels. Side effects included: loss of appetite, gastrointestinal pain, headaches, sedation, dizziness, and vivid dreams.

*Note* AUDIT-C = Alcohol Use Disorders Identification Test-Consumption Questions, BPRS = Brief Psychiatric Rating Scale, CBT = cognitive behavioural therapy, GA = Gamblers Anonymous, GAD-7 = Generalised Anxiety Disorder, GAF = Global Assessment of Functioning Scale, GAS = Goal Attainment Scale, GCS = Gambling Craving Scale, GMA = Gordon Moody Association, G-SAS = Gambling Symptom Assessment Scale, MAAS = Mindful Attention and Awareness Scale, MAT = Meditation Awareness Training, MSSS = Multidimensional Scale of Social Support, NPGC = National Problem Gambling Clinic, NS = not specified, PGSI = Problem Gambling Severity Index, PHQ-9 = Patient Health Questionnaire

### Study design and setting

The most common study design was retrospective chart review, which was used in four studies, while one study employed a retrospective case series, two described single participant case report designs, and one used a cross-sectional survey design. No experimental research designs were identified by our review.

The most common study setting was the gambling treatment clinic setting (described in five studies), with the three remaining studies set in Gamblers Anonymous (GA), residential care settings, or were not specified, respectively.

### Sample characteristics

Recruitment sources were not specified in two articles [24, 27], one study recruited volunteers from an open GA meeting [28], four studies described either prospective or retrospective analyses of clinic patient data collected from either the National Problem Gambling Clinic (NPGC) [29–31] or Gordon Moody Association residential gambling treatment sites [26], and one study conducted a retrospective audit of NPGC case files to identify eligible participants [32].

Across studies, sample size ranged between one [24, 27] and 846 [31], with more males (2,605 of 2,760; 94%) than females studied, and participants’ ages ranged between 17 and 70 years old (M_age_ = 35; SD = 10.37).

#### Problem or harmful gambling status

Participants’ gambling status was not specified in two studies [24, 28], scores on the *Problem Gambling Severity Index* (PGSI) were used to categorise participants in five studies [26, 29–32] and the DSM-IV-TR criteria was employed in one study [27]. Of those studies employing the PGSI, three used a score cut-off of 8+ [30–32], one used a cut-off of 3+ [29], and another did not specify a cut-off [26].

### Gambling specific and other outcomes measures

Most studies measured some form of gambling behaviour either through formal self-report or interview assessment. Gambling-specific cravings were measured in two studies (one of which used the *Gambling Cravings Scale*), self-efficacy and perceived risk in another study, while the *Gambling Symptom Assessment Scale* was employed in another. The PGSI was either a primary or a secondary outcome measure in three of the studies.

Studies employed a range of additional measures, with mental health (anxiety and depression), alcohol and drug use measured by two studies, psychiatric symptoms, global functioning, mindfulness, and goal attainment addressed in one study, treatment completion by two studies, and medication side effects, recovery group identification and social support in one other study, respectively. A further study did not specify any additional measures.

### Narrative details of included studies

Bowden-Jones and George [24] reported the case of an individual with a gambling addiction that had developed following dopaminergic treatment for restless legs syndrome. Findings were narratively described, and it was unclear whether the outcomes were measured using standardised assessments. However, the authors reported how a medication switch to rotigotine and a 10-session CBT treatment resulted in positive client outcomes (client-reported abstinence), although occasional cravings persisted.

Hutchison et al. [28] described a cross-sectional study of the mechanisms underlying the efficacy of GA mutual aid fellowships. Participants were volunteers recruited over three consecutive weeks at fellowship meetings (*n* = 44). Authors reported that identification with a recovery group predicted higher perceived support, higher abstinence self-efficacy, and lower perceived risk in hypothetical gambling-related trigger situations. Analyses further suggested that the relationship between recovery group identification and outcomes (abstinence self-efficacy and perceived risk) were mediated via the provision of social support to group members rather than the receipt of support.

Ronzitti et al. [29] retrospectively analysed NPGC patient data (*n* = 676) over a 2-year period. When comparing smokers and non-smokers at baseline, they found that smokers scored more highly on the *Alcohol Use Disorders Identification Test* (AUDIT-C) and were more likely to have used drugs in the previous 30 days. Statistically significant differences were not found between cohorts’ baseline on PGSI scores or clinical variables (GAD-7 and PHQ-9 scores), and nor was tobacco use related to follow-up PGSI scores, days gambled in the previous month, or other clinical variables.

Ronzitti et al. [31] investigated whether clinical and sociodemographic variables retrospectively predicted dropout among data obtained from a large sample of participants (*n* = 846) attending the NPGC across a three-year period. The authors found that the format of CBT delivery (i.e., group sessions, individual, or a combination) did not predict dropout. However, their analysis suggested that clients who dropped out of treatment were more likely to be single, younger, and unemployed, smoke, and score more highly on the PGSI, GAD-7, and PHQ-9. Ronzitti et al. also found that pre-treatment dropout was associated with younger age and drug use, whilst dropping out during treatment was linked with lower PGSI score, smoking, and having a family history of gambling disorder.

Ronzitti et al. [30] also retrospectively analysed clinic patient data at the NPGC (*n* = 524) to examine the relationship between treatment completion and different gambling activities. They found gaming machine use was a predictor of pre-treatment dropout, while betting on sports events predicted dropping out during treatment.

Roberts et al. [26] retrospectively evaluated predictors of treatment dropout and utilised variables derived from service-specific questionnaires. These included a gambling audit, which provided information on primary forms of gambling engaged in by patients, a need audit, which provided information on mental and physical health, and a life audit, which provided information on significant life events (e.g., divorce, assault during childhood, homelessness). Findings suggested that higher education, higher debts, online gambling, gambling on poker, depression, and adverse childhood experiences predicted dropout. Roberts et al. also found that undertaking shorter treatment programmes (i.e., 3–6 months rather than 9 months) and experiencing previous treatment programmes and medication also predicted dropout rates. Analyses suggested that enforced dropout (compared to voluntary) was predicted by gambling on sports events, smoking, and previous experience of homelessness or depression.

Shonin et al. [27] described a 2-phase treatment comprising second-wave CBT followed by Meditation Awareness Training (MAT) for a patient with a dual diagnosis of schizophrenia and ‘pathological gambling’. They reported moderate improvements during the CBT phase of treatment on the *Brief Psychiatric Rating Scale* (BPRS) and the *Global Assessment of Functioning Scale* (GAF) but no change on the *Gambling Symptom Assessment Scale* (G-SAS) or the *Mindful Attention and Awareness Scale* (MAAS). Following MAT, the authors reported a significant improvement across all outcome variables, with abstinence reportedly being maintained at 3-month follow-up.

Ward et al. [32] investigated the use of naltrexone (an opioid antagonist) as a treatment for non-responders to psychological treatment at the NPGC. Data were collected via a retrospective audit of case files over an 18-month period (*n* = 10). At 6-week follow up, all participants reported reductions in their gambling cravings, with 60% being abstinent during treatment, and a further 20% reducing their gambling to low levels. Side effects reported by patients included a loss of appetite, gastrointestinal pain, headaches, sedation, dizziness, and vivid dreams.

### Funding details and competing interests

Funding information was reported by only two studies. Ward et al. [32] was supported by funding received from the Central and North West London NHS Foundation Trust, while Roberts et al. [26] was funded by the University of Lincoln.

Roberts et al. [26] was the only article in the sample that reported conflicts of interest, reporting various funding sources of the authors over the last three years including GambleAware, the Society for the Study of Addiction, National Institute for Health and Care Research (NIHR), Santander, the Young Gamblers Education Trust, and Cancer Research UK.

## Discussion

Eight gambling treatment studies conducted in the UK met criteria for inclusion in this scoping review. Findings indicated that a range of study designs, recruitment sources, client samples, and analytic approaches were undertaken. Problem or harmful gambling status tended to rely on PGSI scores. Gambling-specific and other outcome measures consisted of gambling behaviour, cravings, abstinence, psychiatric comorbidities, alcohol and substance use, and general wellbeing. This selection of studies was small, somewhat variable in focus and outcomes, and most notably, did not include any empirical evaluations of treatments (e.g., randomised control trials, RCTs) recommended for harmful gambling.

Our review found no evidence that interventions for harmful gambling have been tested using experimental research designs, such as RCTs [33, 34] in a UK context. The overreliance on either retrospective chart reviews (employing secondary analysis of existing data) or single-subject case reports indicate that treatment-oriented researchers may lack the resources necessary to conduct large-scale, direct comparison of the effectiveness of different treatments for gambling-related problems. This observation contrasts with the stated aims of the present development of NICE guidelines for the treatment of harmful gambling to identify what treatments work for whom and in what settings. The framework provided by RCTs contribute to the empirical basis of treatment effectiveness relative to other conditions, such as treatment as usual or waitlist control groups, and are a prominent feature of the gambling treatment literature in other countries and jurisdictions. For instance, a growing number of RCTs and other treatment-focused studies have emerged from Germany evaluating its free of charge outpatient addiction care facilities service [35] and self-guided internet-based interventions [33] Given the similarities between the gambling landscape and treatment service sectors in both Germany and the UK, one might expect comparable levels of published intervention research but that is clearly not yet the case. While it is beyond the remit of the present scoping review to speculate as to the potential reasons for this absence of published treatment studies, it would be salutary to identify the main characteristics of gambling based RCTs from other countries and the role of legislative, professional, or funding background in determining the treatment choices available to people seeking help with harmful gambling.

Our findings confirmed a paucity of gambling treatment outcome studies employing single case experimental designs (SCED) [36, 37]. Often referred to as “N-of-1” designs or “single-patient trials”, SCEDs measure the behaviour of one or more individuals across extended time intervals in the presence and absence of the independent variable [38, 39]. These designs permit repeated measurement of the effects of experimental manipulations or the introduction and withdrawal of an intervention without the requirement to randomise or exclude participants receiving different therapies or with disorder-specific comorbidities. Single-case experimental designs are considered level 1 A evidence for treatment decision purposes by the Oxford Centre for Evidence-Based Medicine [40], are recommended by the Medical Research Council [41] and the What Works Clearinghouse panel [42, 43], and their use in behavioural research is supported through guidelines produced by the CONsolidated Standards Of Reporting Trials (CONSORT) Extension for N-of-1 Trials (CENT) [39, 44] and the Single-Case Reporting guideline In BEhavioral interventions (SCRIBE) [45] endorsed by the American Psychological Association [46]. While there are examples of their effective use in intervention [47] and lab-based analogue studies [48, 49,50], it appears that the adoption of SCEDs in gambling treatment studies generally and in the UK specifically has been slow. It would be helpful therefore for future research to consider the relative merits of SCEDs for gambling treatment studies and address any perceived barriers and obstacles to their wider uptake and dissemination.

Participants’ gambling status was determined using either the PGSI, DSM, or was not specified. In some cases, gambling status was inferred from PGSI scoring criteria of 8 or above, while other studies either adopted lower PGSI criteria or none were stated. The diagnostic accuracy and validity of the PGSI at identifying problem and at-risk gambling is well established [51,52] and our findings highlight its widespread use in UK gambling treatment research. While factors such as resource demand and other constraints on clinical practice may sustain the continued use of the PGSI as a measure of gambling harm and severity, it is important to remember that it does not provide a formal diagnosis of gambling disorder. That status is reserved for DSM-5 or ICD-11 based instruments and it was noteworthy that only one included study [27] had employed the then-current DSM-IV-TR based diagnosis of ‘pathological gambling’. Thus, there is a need for contemporary screening and diagnostic instruments for ICD-11 and DSM-5 gambling disorder [28, 29] to enable further gambling treatment research. These issues clearly warrant research attention [24].

Included studies rarely specified participant recruitment methods, which is perhaps unsurprising as the majority constituted secondary analyses or audits of existing clinic data and did not necessitate recruitment of new participants. Of the three studies that recruited participants, two did not state recruitment details and one study sought help-seeking volunteers from an onsite GA meeting [30]. It is striking therefore that the extant UK gambling treatment literature primarily recruited from one clinic (the NPGC) or residential settings (e.g., Gordon Moody Association), with no evidence of recruitment from across the wider NGSN, third sector/charitable agencies, or online settings. This finding may cast doubt as to the representativeness of the UK gambling treatment literature. Given the wide range of gambling treatment and support options available in the UK, recruiting help-seeking individuals from multiple settings would greatly benefit the identification of what treatment works best and for whom. In the absence of evidence of wider recruitment, the potential barriers to a more representative gambling treatment landscape in the UK may include lack of a critical mass of clinically oriented researchers, difficulties accessing clinical populations, limited funding opportunities, and competing research priorities. Notwithstanding these important factors, it is possible also that the gambling treatment research field in the UK is relatively underdeveloped and has not yet had sufficient time to foster the collaborative networks required for work of this kind with relevant stakeholders. Future reviews should therefore update and assess progress made towards overcoming these barriers and in meeting research priorities aimed at wider, more representative recruitment in gambling treatment research and evaluation studies [24].

Two of the included studies investigated co-occurring depression and anxiety with the PHQ and GAD-7, respectively [31–33]. Among individuals with a diagnosis of pathological or disordered gambling, mood disorders and anxiety disorders are two of the most highly comorbid psychiatric disorders, along with substance-related disorders and impulse-control disorders [53, 54]. Given this high comorbidity, it would have been helpful for more studies to have assessed mood and anxiety disorders among individuals seeking treatment for gambling. Instead, the existing work from the two included studies constituted a retrospective analysis of trends in client profiles from one clinic. Ronzitti et al. [31] found that individuals with gambling disorder who used tobacco did not differ from nonusers in anxiety (GAD-7), depression (PHQ-9), or gambling severity scores, while another study the same authors [33] found that individuals who dropped out either pre-treatment or during treatment had higher anxiety, depression, and gambling severity scores than those who completed treatment, and were more likely to use tobacco. Clearly, future research and treatment evaluation studies should incorporate assessment of potential comorbid disorders and systematically analyse harmful gambling risk factors among both the general population and those who participate in harmful gambling [6]. Future studies might also consider screening for a wider range of potential comorbidities such as impulse-control disorders [54], autism [55] and trauma [56].

Evaluating the efficacy of specific treatments was not always the aim of the included studies, yet it was notable that participants in six of the eight articles were receiving CBT as either a primary intervention or as part of a treatment package. Cognitive-behavioural therapy (CBT) is widely employed to treat harmful or problematic gambling [57-60], and our findings provide evidence of its continued use within UK treatment settings. Although evidence suggests that CBT may more effective over the short term (0–3 months post-treatment) rather than the long term (9–12 months) [61], it is readily integrated with other therapeutic approaches targeting more sustained behaviour change [62–65]. Our review, however, found no evidence of the incorporation of CBT with other psychological interventions and thus we are unable to draw any conclusions as to its effectiveness for people who participate in harmful gambling. Our findings therefore chime with calls for more randomized controlled trials on psychological interventions for gambling disorder [24, 60]. Clearly, there is a need for further evaluation studies of the effectiveness of CBT, and components of CBT, with and without additional intervention, with people in the UK who participate in harmful gambling.

The general absence of experimental gambling research treatment studies was also reflected both in the lack of formal power analyses for estimated sample size and any other open science practices among the included studies. In their recent scoping review of open science practices in gambling research, Louderback et al. [66] noted that 6.4% (*n* = 32) of 500 studies published between 2016 and 2019 included a power analysis. Gambling industry-funded studies were more likely to include a power analysis, particularly in experimental studies. This suggests that experimental research studies reported power analyses to ensure robust statistical inferences and that the absence of such analyses from observational studies indicated a lower prioritizing of statistical power (despite the often-large sample sizes reported in these studies). However, as our findings did not include any experimental studies, the absence of power analysis and any consideration of the effect size of findings is rather moot [66, 67]. All other open science practices such as preregistration and open data sharing were notably absent from the present set of included studies, suggesting a great deal more needs to be done to increase the uptake of open science in gambling research and treatment studies.

The finding of Louderback et al. [66] that gambling industry-funded experimental studies were more likely to include a power analysis may indicate a greater need for perceived objectivity given the controversial nature of the role of industry funding in gambling research. While our findings cannot directly speak to this issue given, we only identified non-experimental, UK-based treatment studies, only two of the eight studies listed funding details [26, 34], and of these only Roberts et al. [26] reported conflicts of interest and receipt of previous sources of funding. One must therefore assume that the remaining studies either did not obtain funding or the reporting requirements of the journals did not specify providing the funding source. The role of funding source and potential for conflicts of interest in gambling research is understandably an important and growing research focus [67, 69–72]. To date, analyses have shown that gambling industry-funded research is more likely than non-industry funded research to report potential conflicts of interest [73]. Similarly, studies funded by the gambling industry are not more likely than studies funded by other sources to report either confirmed, partially confirmed, or rejected hypotheses and predictions. In a follow-up assessment of study designs used in responsible gambling research between studies funded by the gambling industry or not, Ladouceur et al. [70] found no difference between funding source and study design characteristics such as outcome measures and the use of comparator groups. This notwithstanding, our findings add support to calls that all journals publishing gambling work should include a statement of competing interests and the role, if any, of funding sources [69–73].

Our scoping review is not without limitations. We did not include a risk of bias assessment as it is not standard practice in scoping reviews to critically appraise identified sources of evidence [19]. However, it may have been helpful to do so to show how robust each included study was in terms of key methodological and reporting requirements. In any event, the small number of included studies would preclude a more formal examination of potential bias. Also, we limited our search to the UK-based treatment literature as we were interested in scoping this work for the first time, given the ongoing development of the NICE guidelines on treatments for harmful gambling. As such, we inevitably excluded comparable studies from other countries with differing policy and legislative frameworks surrounding gambling treatment. It would thus be useful for future work to scope the global literature and to identify common practices or gaps in the treatment of harmful gambling [10, 60].

## Conclusions

In conclusion, the present scoping review highlighted a paucity of gambling treatment research conducted in UK settings. Our findings support calls made by Bowden-Jones et al. [24] for an urgent need to set new research priorities to support the treatment of harmful gambling. To this, we would add that the objective identification of potential barriers and obstacles to wider and more intensive gambling treatment research in the UK also warrants attention. While it is not the place of the present scoping review to speculate as to this relative dearth of research, some authors have argued that the absence of a statutory levy on industry profits administered by an independent authority may have impeded UK based gambling treatment research through diminished funding opportunities [68]. Indeed, jurisdictions with levies or hypothecated taxation on gambling industry profits, such as Australia, may have produced comparatively more treatment research studies than the UK [e.g., 74, 75]. Clearly, further systematic analysis of international gambling treatment research is warranted [10, 59]. Conducting treatment evaluation research anywhere however is expensive, time-consuming, and requires collaboration across multiple sectors. It is worth highlighting that although such work may at present be supported by a diverse range of funders (e.g., UKRI, NIHR, local government, charities, etc.), having access to annual levy funds may help support further growth capacity in UK gambling treatment research.

### Electronic supplementary material

Below is the link to the electronic supplementary material.

Supplementary Material 1

Supplementary Material 2

## Data Availability

The datasets generated and analysed during the current study are available in the OSF repository at https://osf.io/6wbdm/.
